# The Cannabigerol Derivative VCE-003.2 Exerts Therapeutic Effects in 6-Hydroxydopamine-Lesioned Mice: Comparison with The Classic Dopaminergic Replacement Therapy

**DOI:** 10.3390/brainsci13091272

**Published:** 2023-08-31

**Authors:** Santiago Rodríguez-Carreiro, Elisa Navarro, Eduardo Muñoz, Javier Fernández-Ruiz

**Affiliations:** 1 Instituto Universitario de Investigación en Neuroquímica, Departamento de Bioquímica y Biología Molecular, Facultad de Medicina, Universidad Complutense, 28040 Madrid, Spain; santirod@ucm.es (S.R.-C.); elisnava@ucm.es (E.N.); 2 Centro de Investigación Biomédica en Red de Enfermedades Neurodegenerativas (CIBERNED), 28029 Madrid, Spain; 3 Instituto Ramón y Cajal de Investigación Sanitaria (IRYCIS), 28034 Madrid, Spain; 4 Instituto Maimónides de Investigación Biomédica de Córdoba (IMIBIC), 14004 Córdoba, Spain; fi1muble@uco.es; 5 Departamento de Biología Celular, Fisiología e Inmunología, Universidad de Córdoba, 14004 Córdoba, Spain; 6 Hospital Universitario Reina Sofía, 14004 Córdoba, Spain

**Keywords:** VCE-003.2, L-DOPA/benserazide, Parkinson’s disease, 6-OHDA-lesioned mice, cannabinoids, PPAR-γ

## Abstract

(1) Background: A cannabigerol aminoquinone derivative, so-called VCE-003.2, has been found to behave as a neuroprotective agent (administered both i.p. and orally) in different experimental models of Parkinson’s disease (PD) in mice. These effects were exerted through mechanisms that involved the activation of a regulatory site within the peroxisome proliferator-activated receptor-γ (PPAR-γ). (2) Methods: We are now interested in comparing such neuroprotective potential of VCE-003.2, orally administered, with the effect of the classic dopaminergic replacement therapy with L-DOPA/benserazide in similar conditions, using 6-hydroxydopamine-lesioned mice. (3) Results: The oral administration of VCE-003.2 during 14 days at the dose of 20 mg/kg improved, as expected, the neurological status (measured in motor tests) in these mice. This correlated with a preservation of TH-labelled neurons in the substantia nigra. By contrast, the treatment with L-DOPA/benserazide (during 7 days at 2 mg/kg) was significantly less active in these experimental conditions, in concordance with their profile as a mere symptom-alleviating agent. (4) Conclusions: Our results confirmed again the therapeutic profile of VCE-003.2 in experimental PD and revealed a different and more relevant effect, as a disease modifier, compared to the classic symptom-alleviating L-DOPA treatment. This reinforces the interest in VCE-003.2 for a future clinical development in this disease.

## 1. Introduction

Cannabinoids are pleiotropic compounds that, acting through multiple pharmacological targets within the endocannabinoid system, as well as in other signaling systems, have demonstrated to be promising neuroprotective agents [1]. Such neuroprotective potential has been preclinically investigated in different neurological conditions, such as accidental brain damage (e.g., stroke and brain trauma) and chronic progressive disorders (e.g., Alzheimer’s disease, amyotrophic lateral sclerosis, and Huntington’s disease (HD)) [2]. One of the disorders that has been more investigated to date is Parkinson’s disease (PD). In this disease, cannabinoid-based therapies may serve to delay disease progression, but also to alleviate specific parkinsonian symptoms [3,4]. Thus, preclinical studies have demonstrated that modulating the cannabinoid receptor type 1 (CB_1_) may serve for reducing parkinsonian signs such as bradykinesia and immobility [5], tremor [6] and/or L-DOPA-induced dyskinesia [7], This can occur even combined in combination with other agents, such adenosine receptor ligands [8,9] such as these receptors may form heteromers with the CB_1_ receptor resulting in possible synergic effects at a pharmacological level [10,11]. Such a combination between CB_1_ and adenosine receptor ligands has been already investigated in pathological conditions other than PD [12,13].

Modulating the CB_1_ receptor may also serve to afford neuroprotective effects in PD [14,15]. However, most cannabinoids proposed as neuroprotectant agents in this disease target the cannabinoid receptor type 2 (CB_2_), whose activation entails anti-inflammatory effects in different PD models [16,17,18,19]. A similar effect has been found with cannabinoids that activate the peroxisome proliferator-activated receptor (PPAR)-γ [20,21] and even that target GPR55, an orphan receptor that has been recently associated with the endocannabinoid system [22,23]. Lastly, the neuroprotective effects in PD of some specific phytocannabinoids (e.g., cannabidiol) have been assigned to receptor-independent antioxidant effects [24] or even associated to activity as modulators of the antioxidant transcription factor Nrf2 [25]. Therefore, these studies have placed several cannabinoid compounds in a promising position for serving to generate a cannabinoid-based therapy for specific symptoms and, in particular, for disease progression in patients affected by PD.

An interesting cannabinoid compound in PD is the non-thiophilic CBG quinone derivative VCE-003.2, whose chemical structure, mechanism of action and other characteristics (in comparison with CBG, its original naturally-occurring phytocannabinoid) have been previously published [20,26,27]. VCE-003.2 behaves as a PPAR-γ activator with no activity at the CB_1_/CB_2_ receptors [26]. This study investigated the effect of VCE-003.2 in murine models of HD, confirming its neuroprotectant profile exerted by activating PPAR-γ and its ability to cross the blood–brain barrier after systemic administration. Moreover, VCE-003.2, given orally, was found to also be neuroprotective and to induce neurogenesis in experimental HD [28]. As regards to experimental models of PD, VCE-003.2, given i.p. or orally, has been found to be active as an anti-inflammatory and neuroprotective agent against inflammation-driven neuronal deterioration in LPS-lesioned mice [20,21]. It was also active in 6-hydroxydopamine (6-OHDA)-lesioned mice [29], a model characterized by mitochondrial dysfunction and oxidative stress. These effects were found to be mediated by its binding at a functional alternative site (different from the canonical binding site used by glitazones) in the PPAR-γ receptor, as revealed in in vitro studies carried out in cell-based assays [20,29].

We wanted now is to further explore the neuroprotective potential of VCE-003.2, using an oral formulation, comparison with the reference therapy in PD using L-DOPA/benserazide [30]. Such a comparison is a necessary step in the development of any new antiparkinsonian agent, in this case VCE-003.2, towards its clinical exploitation in PD. However, it is important to remark that such dopaminergic replacement therapy with L-DOPA/benserazide was approved to alleviate specific parkinsonian symptoms (e.g., akinesia and rigidity) in PD patients [30]. By contrast, it is generally accepted that L-DOPA/benserazide has no effect on disease progression, despite a few studies that reported certain neuroprotective effects in experimental models [31,32] pending further confirmation. To this end, our experimental design was aimed at evaluating the neuroprotective effect of a chronic administration of VCE-003.2 given orally to 6-OHDA-lesioned mice for 14 days, which was compared with the effect of a chronic administration of L-DOPA/benserazide given i.p. for 7 days (to use a shorter treatment was necessary to diminish the occurrence of dyskinesia [33]). The efficacy of both treatments was determined with motor tests and immunostaining for tyrosine hydroxylase (TH) and glial markers (GFAP and CD68) in the substantia nigra. It is important to remark that all analyses, but this is particularly important in the case of behavioral tests, were carried out at 24 h from the last injection. This means that the possible behavioral effects found should be related more to neuroprotection rather than to symptom alleviation, which would be visible only at shorter times (1–2 h).

## 2. Materials and Methods

### 2.1. Animal and Surgical Lesions

Male C57BL/6 mice were housed in our animal facilities (CAI-Animalario, Faculty of Medicine, Complutense University, ref. ES280790000086) under controlled photoperiod (08:00–20:00 light) and temperature (22 ± 1 °C), and with free access to standard diet and water. They were used at adult age (6–8 month-old; 24–30 g weight) for experimental purposes. All experiments were conducted according to national and European guidelines (directive 2010/63/EU), as well as conforming to ARRIVE guidelines, and were approved by the “Comité de Experimentación Animal” of our university (PROEX: 201.8/22). Mice were anaesthetized (ketamine 40 mg/kg + xylazine 4 mg/kg, i.p. purchased from Sigma-Aldrich, Madrid, Spain). This was followed by pretreatment with desipramine (25 mg/kg, i.p., purchased from Sigma-Aldrich, Madrid, Spain) just 30 min before mice received 6-OHDA free base (2 μL at a concentration of 2 μg/μL saline in 0.02% ascorbate to avoid oxidation; both purchased from Sigma-Aldrich, Madrid, Spain) or saline (for control mice) injected stereotaxically into the right striatum at a rate of 0.5 μL/min. To this end, we used the following coordinates: +0.4 mm AP, ±1.8 mm ML and −3.5 mm DV, as described in Alvarez-Fischer et al. [34]. Once injected, the needle was left in place for 5 min before being slowly withdrawn. This avoided generating reflux and a rapid increase in intracranial pressure. Control animals were sham-operated and injected with 2 μL of saline using the same coordinates. After the application of 6-OHDA or saline, mice were subjected to pharmacological treatments as described in the following section. The lesions were generated using unilateral injection with the contralateral structures serving as controls for the different analyses.

### 2.2. Pharmacological Treatments and Sampling

After the application of 6-OHDA, animals were treated with the following compounds: (i) VCE-003.2 (provided by Emerald Health Pharmaceuticals, San Diego, CA, USA) given orally at a dose of 20 mg/kg according to previous studies [29], initiating the treatment at 24 h after the lesion and daily repeating during 14 days; (ii) L-DOPA (Sigma-Aldrich Chem., Madrid, Spain) and benserazide (Sigma-Aldrich Chem., Madrid, Spain), given i.p. at the dose of 2 mg/kg in both cases according to previous studies [33], initiating the treatment 7 days after the lesion and daily repeating during 7 additional days (as indicated before, the use of a shorter treatment was necessary to diminish the occurrence of dyskinesia [33]); or (iii) the vehicle for VCE-003.2 (sesame oil) given orally during 14 days (50% of mice in this group) and the vehicle for L-DOPA/benserazide (0.9% saline) given i.p. during 7 days (remaining 50% of animals). At the end of the treatment (24 h after the last injection), mice were analysed in different behavioural tests just before being killed by rapid and careful decapitation. Their brains were rapidly removed and fixed for one day at 4 °C in fresh 4% paraformaldehyde (Sigma-Aldrich, Madrid, Spain) prepared in 0.1 M PBS, pH 7.4. Samples were cryoprotected by immersion in a 30% sucrose (Sigma-Aldrich, Madrid, Spain) solution for 48 h, and finally stored at −80 °C for immunohistochemical analysis in the substantia nigra.

### 2.3. Behavioural Tests

Cylinder rearing test (CRT). Given that the lesions were unilateral, this test attempts to quantify the degree of forepaw (ipsilateral, contralateral, or both) preference for wall contacts after placing the mouse in a methacrylate transparent cylinder (diameter: 15.5 cm; height: 12.7 cm; [35]). Each score was made from a 3 min trial with a minimum of 4 wall contacts.

Pole test. Mice were placed head upward on the top of a vertical rough-surfaced pole (diameter 8 mm; height 55 cm) and the time until animals descended to the floor was recorded with a maximum duration of 90 s. When the mouse was not able to turn downward and instead dropped from the pole, the time was taken as 90 s (default value).

### 2.4. Immunohistochemical Procedures

Brains were sliced in coronal sections (30 µm thick; containing the substantia nigra) in a cryostat (Leica Biosystems, Wetzlar, Germany) and collected on antifreeze solution (glycerol/ethylene glycol/PBS; 2:3:5) and stored at −20 °C until used. Brain sections were mounted on gelatin-coated slides, and once adhered, washed in 0.1 M potassium PBS (KPBS) at pH 7.4. Endogenous peroxidase was blocked by 30 min incubation at room temperature in peroxidase blocking solution (Dako Cytomation, Glostrup, Denmark). After several washes with KPBS, sections were incubated overnight at room temperature with the following polyclonal antibodies: (i) rabbit anti-mouse TH (Chemicon-Millipore, Temecula, CA, USA) used at 1:200; (ii) rat anti-mouse CD68 antibody (AbD Serotec, Oxford, UK) used at 1:200; or (iii) rabbit anti-mouse GFAP antibody (Dako Cytomation, Glostrup, Denmark) used at 1:200. Dilutions were carried out in KPBS containing 2% bovine serum albumin and 0.1% Triton X-100 (both purchased in Sigma-Aldrich, Madrid, Spain). After incubation, sections were washed in KPBS, followed by incubation with the corresponding biotinylated secondary antibody (1:200) (Vector Laboratories, Burlingame, CA, USA) for 1 h at room temperature. Avidin–biotin complex (Vector Laboratories, Burlingame, CA, USA) and 3,3′-diaminobenzidine substrate–chromogen system (Dako Cytomation, Glostrup, Denmark) were used to obtain a visible reaction product. Negative control sections were obtained using the same protocol with omission of the primary antibody. A Leica DMRB microscope and a DFC300FX camera (both from Leica Biosystems, Wetzlar, Germany) were used for the observation and photography of the slides, respectively. For quantification of TH, CD68, or GFAP immunostaining in the substantia nigra, we used the NIH Image Processing and Analysis software (ImageJ; NIH, Bethesda, MD, USA) using 4–5 sections, separated approximately by 200 µm, and observed with a 10× objective. In all sections, the same areas of the substantia nigra were analyzed. The analyses were always conducted by experimenters who were blinded to all animal characteristics. Data were expressed as percentage of immunostaining intensity in the ipsilateral (lesioned) side over the contralateral (non-lesioned) side.

### 2.5. Statistics

Data were assessed using one-way ANOVA, followed by the Tukey test using GraphPad Prism, version 8.00 for Windows (GraphPad Software, San Diego, CA, USA). A *p* value lower than 0.05 was used as the limit for statistical significance. The sample sizes in the different experimental groups for both behavioral and histopathological analyses were always ≥5 (exact sample sizes are visible in the scatter plots presented in the figures). In the case of the groups treated with vehicle, there was not any significant difference between the values in the two subgroups (sesame oil given orally during 14 days and saline given i.p. during 7 days) for both behavioral and histopathological data, so they were combined for statistical analysis and presentation. 

## 3. Results

**Figure 1:**
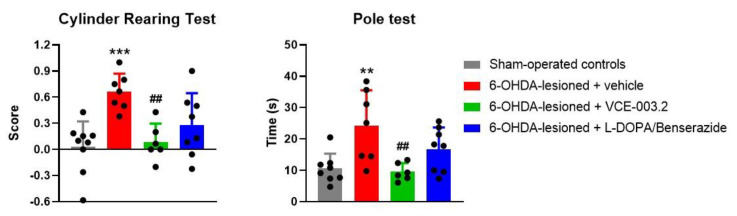
Response in the cylinder rearing and pole tests of 6-OHDA-lesioned mice treated with VCE-003.2 (at an oral dose of 20 mg/kg), L-DOPA/benserazide (at an i.p. dose of 2 mg/kg) or vehicle (sesame oil or 0.9% saline, respectively), and the corresponding controls. Data corresponded to 24 h after the last dose of VCE-003.2 (2 weeks of daily treatment) or L-DOPA (1 week of daily treatment). Values are mean ± SD and were analyzed by one-way ANOVA followed by the Tukey test (** *p* < 0.01, *** *p* < 0.005 versus vehicle-treated sham mice; ## *p* < 0.01 versus vehicle-treated 6-OHDA-lesioned mice).

In this study, we first pursued to confirm whether an oral formulation of VCE-003.2 in sesame oil at 20 mg/kg (two weeks of daily treatment) is active as a neuroprotectant in 6-OHDA-lesioned mice, as previously found in this [29] and other experimental models [20,21] of PD. Thus, our data indicated the occurrence of motor (deteriorated animal performance in the pole test and the CRT) and histopathological (loss of TH-positive neurons in the substantia nigra) abnormalities in 6-OHDA-lesioned mice to an extent similar to the data described in previous studies using this model [29]. These motor and histopathological abnormalities were again attenuated after the oral administration of VCE-003.2, to an extent similar to the previous data obtained in this model [29] and also in additional models [20,21]. These beneficial effects were evident, in particular, in the animal response in the CRT, as the elevation in the score of 6-OHDA-lesioned mice that reflects hemiparesis was significantly reversed by the treatment with oral VCE-003.2 (F(3,29) = 7.42, *p* < 0.001; Figure 1). Similar changes were seen in the pole test, with a total reversion of the elevated time to descend the pole after the treatment with oral VCE-003.2 (F(3,28) = 6.01, *p* < 0.005) (Figure 1).

**Figure 2:**
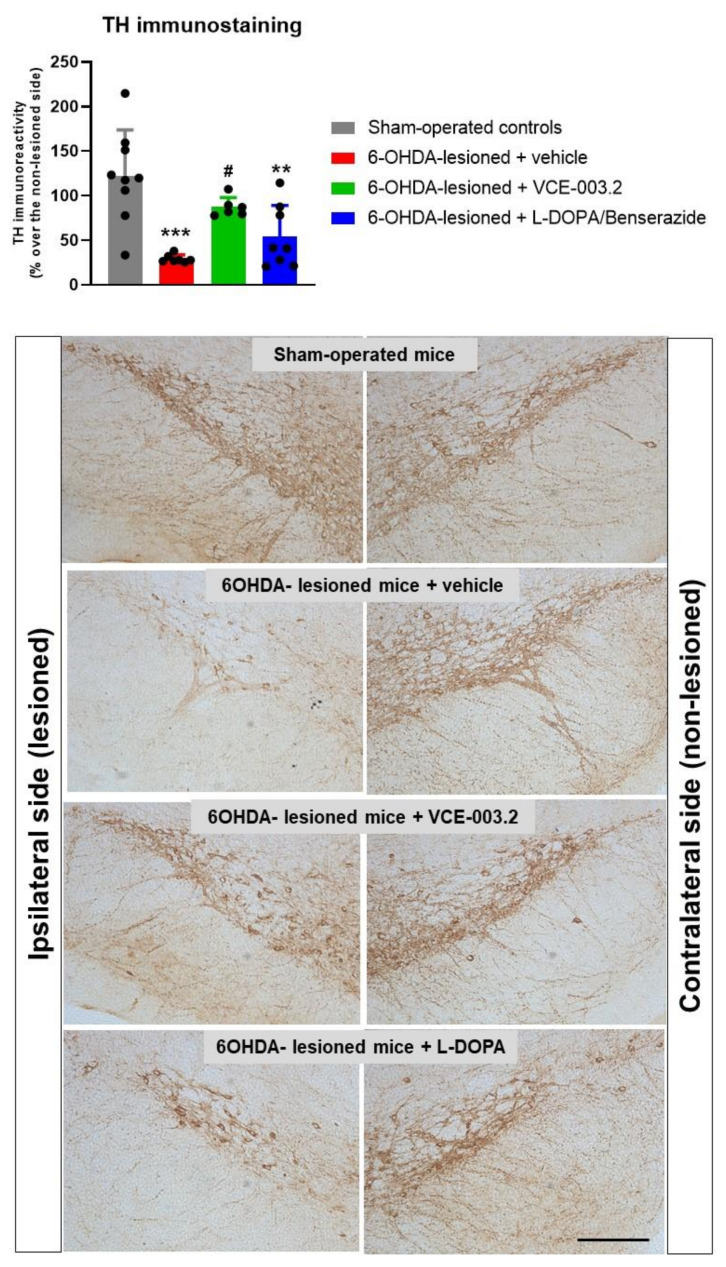
TH immunoreactivity levels (expressed as % over the contralateral non-lesioned side) in the substantia nigra of 6-OHDA-lesioned mice treated with VCE-003.2 (at an oral dose of 20 mg/kg), L-DOPA/benserazide (at an i.p. dose of 2 mg/kg) or vehicle (sesame oil or 0.9% saline, respectively), and the corresponding controls. The figure also includes representative microphotographs of ipsi-lateral lesioned and contralateral non-lesioned sides for each experimental group (scale bar = 200 µm). Data corresponded to 24 h after the last dose of VCE-003.2 (2 weeks of daily treatment) or L-DOPA (1 week of daily treatment). Values are mean ± SD, and were analyzed by one-way ANOVA followed by the Tukey test (** *p* < 0.01, *** *p* < 0.005 versus vehicle-treated sham mice; # *p* < 0.05 versus vehicle-treated 6-OHDA-lesioned mice).

These neurological benefits elicited by VCE-003.2 were associated with an apparent preservation of nigrostriatal neurons as detected using TH immunostaining in the substantia nigra (Figure 2). Thus, TH immunoreactivity levels in 6-OHDA-lesioned mice were strongly reduced compared to the control mice, but this reduction was significantly reversed by the chronic treatment with oral VCE-003.2 (F(3,29) = 11.31, *p* < 0.0001; Figure 2). It is true that our analysis only detected immunoreactivity levels for TH and that this is not necessarily a confirmation of more TH-positive cells. However, the morphological analysis of TH-immunostained sections in the different experimental groups appears to confirm the existence of more TH-immunolabelled cells in VCE-003.2-treated mice.

As indicated in the Introduction, we also pursued to compare the efficacy of VCE-003.2 as an antiparkinsonian agent with the effects of L-DOPA/benserazide (2 mg/kg, i.p., one week of daily treatment following the method described in Lundblad et al. [33]). This is the reference dopaminergic replacement therapy approved for PD patients, but this therapy is, in general, not active as a disease modifier being exclusively addressed to alleviate certain parkinsonian signs [30]. Thus, our data confirmed that the treatment with L-DOPA/benserazide only caused very modest effects (without reaching statistical significance compared to vehicle-treated 6-OHDA-lesioned mice) in the CRT and the pole test (Figure 1), as well as in TH immunostaining (Figure 2), in concordance with the notion that it only serves as a symptom-alleviating agent. Such an observation discards the data published previously in a few studies that suggest a certain neuroprotective activity for L-DOPA/benserazide too [31,32]. In addition, it demonstrates that the expected benefits of L-DOPA/benserazide against parkinsonian symptoms (without preservation of TH-positive neurons) were not visible here, as the response in the motor tests was analyzed at 24 h after the last injection, and not at shorter times (1–2 h) when they should be much more visible.

**Figure 3:**
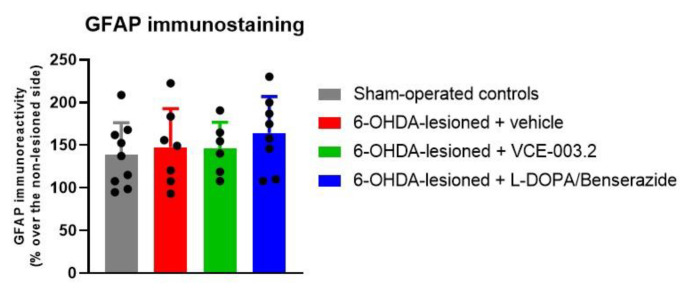
GFAP immunoreactivity levels (expressed as % over the contralateral non-lesioned side) in the substantia nigra of 6-OHDA-lesioned mice treated with VCE-003.2 (at an oral dose of 20 mg/kg), L-DOPA/benserazide (at an i.p. dose of 2 mg/kg) or vehicle (sesame oil or 0.9% saline, respectively), and the corresponding controls. Data corresponded to 24 h after the last dose of VCE-003.2 (2 weeks of daily treatment) or L-DOPA (1 week of daily treatment). Values are mean ± SD and were analyzed by one-way ANOVA followed by the Tukey test.

**Figure 4:**
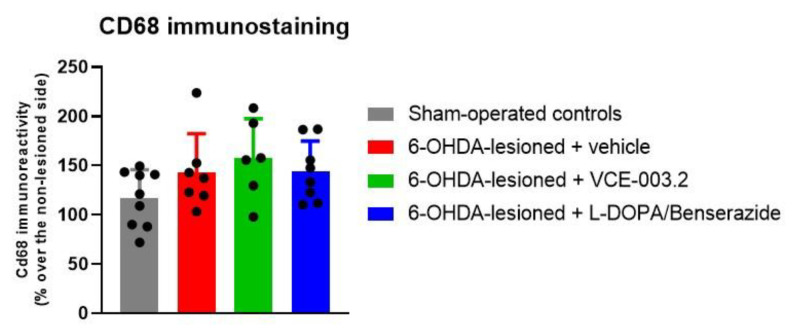
Cd68 immunoreactivity levels (expressed as % over the contralateral non-lesioned side) in the substantia nigra of 6-OHDA-lesioned mice treated with VCE-003.2 (at an oral dose of 20 mg/kg), L-DOPA/benserazide (at an i.p. dose of 2 mg/kg) or vehicle (sesame oil or 0.9% saline, respectively), and the corresponding controls. Data corresponded to 24 h after the last dose of VCE-003.2 (2 weeks of daily treatment) or L-DOPA (1 week of daily treatment). Values are mean ± SD and were analyzed by one-way ANOVA followed by the Tukey test.

Lastly, our study also included immunohistochemical analysis of glial reactivity. However, according to the data described in our previous study [29], the elevation of immunoreactivity levels for the astrocyte marker GFAP (F(3,29) = 0.62, ns) and for the microglial marker CD68 (F(3,29) = 1.88, ns) was modest (or did not exist) with this 6-OHDA lesion, counteracting the determination of whether VCE-003.2 or L-DOPA/benserazide may be active at this level (Figure 3 and Figure 4).

## 4. Discussion

Our study is a new step in the process to move the phytocannabinoid derivative VCE-003.2 towards the clinical scenario as a potential neuroprotective therapy for PD patients. The first step in this process was to demonstrate the efficacy of VCE-003.2 as neuroprotective agent in different in vivo models of PD that recapitulate the different pathogenic events in this disease. Thus, we demonstrated that VCE-003.2 was active against inflammatory events eliciting neuronal injury (LPS model) [20,21], as well as against mitochondrial dysfunction and oxidative stress (6-OHDA model) [20,29] and could also be effective against α-synuclein dysregulation and aggregation (work in progress). This was followed, in a second step, by the confirmation that VCE-003.2 was also active not only after i.p. administration, but also when given orally (LPS model) [21], which facilitates its formulation for administration in patients.

Now, a necessary new step is to compare the efficacy of VCE-003.2 with the effect in the same conditions of the classic dopaminergic replacement therapy with L-DOPA/benserazide licensed for PD patients. It is important to remark, as indicated in the Introduction, that L-DOPA/benserazide treatment is addressed to attenuate specific parkinsonian symptoms (e.g., akinesia, rigidity, and postural instability), not to slow disease progression. However, this comparison is necessary given that this dopaminergic replacement treatment is the reference therapy approved in PD patients [28]. In this sense, any new antiparkinsonian agent needs to confirm that it offers advantages, for example, more potent effects, less side effects, or activity at areas not covered by the approved therapy (the latter is the option investigated here). It was also necessary to confirm, or not, the data provided by a few studies that reported certain neuroprotective activity in experimental PD models with this dopaminergic replacement therapy [31,32]. Thus, according to the data obtained in the study presented here, we can confirm that VCE-003.2 was also active given orally in 6-OHDA-lesioned mice and presented a neuroprotective effect that was not found with L-DOPA/benserazide. This was seen in the animal response in the two motor tests analyzed here, in which the effects of VCE-003.2 almost completely reversed the motor deficiencies. Nevertheless, L-DOPA/benserazide only showed very small trends towards a reversion that were not statistically significant compared to lesioned animals treated with the vehicle. It is likely that these modest effects only reflect the symptom-alleviating effect of L-DOPA/benserazide. In addition, its small magnitude may be caused because the motor tests were carried out at 24 h after the last administration, and not during the acute period (e.g., 1–2 h) where this combination should be more active as a symptomatic treatment. By contrast, VCE-003.2 showed a complete reversion seen at the behavioral level, which should be caused by its capability to preserve nigral TH-positive neurons. In the case of L-DOPA/benserazide, such capability was not evident at the statistical level, as already indicated in previous studies [31,32]. This different response is possibly associated with the molecular mechanisms that are activated by each of these two treatments. In the case of L-DOPA/benserazide, the mechanism would be the recovery of dopamine levels enabling only symptom-alleviating effects [30]. In the case of VCE-003.2, the mechanism would be the activation of PPAR-γ signaling, which would result in the preservation of TH-positive neurons, as demonstrated in previous studies [20,21]. It is also important to remark that PPAR-γ receptors have been already found to serve as a neuroprotective target in PD when activated by non-cannabinoid compounds (e.g., glitazones) [36]. Therefore, this is possibly the major difference between the two treatments investigated here, which situates PPAR-γ as a relevant protein that may work as a promising target to develop a cannabinoid-based neuroprotective therapy.

Another aspect of our study that requires some discussion is the lack of any changes in astroglial and microglial reactivity with this 6-OHDA lesion. This avoids to see whether the treatment with VCE-003.2 was effective against this pathogenic event as found in PD models in which inflammation drives neuronal deterioration [20,21]. Our observation here is likely associated with the fact that glial reactivity in the 6-OHDA model is relatively residual and always secondary to neuronal injury, as described in previous studies [18,29].

## 5. Conclusions

Our data confirmed that 6-OHDA-lesioned mice exhibited an altered response in the cylinder rearing and pole tests, recapitulating some PD-like neurological signs. These responses were provoked by an intense loss of nigral dopaminergic neurons. The treatment with an oral dose of VCE-003.2 (20 mg/kg) avoids the deterioration of animal performance in the two motor tests, and such a benefit was associated with a parallel preservation of nigral TH-containing neurons. All these beneficial effects were not seen (or were resulted to be mere trends) after the treatment with L-DOPA/benserazide, then revealing the advantages of VCE-003.2 against the standard L-DOPA/benserazide therapy in PD. This finding will require further confirmation in future studies aimed at exploring VCE-003.2 administered with: (i) L-DOPA/benserazide to optimize the combination of disease modifying and symptom-alleviating effects; (ii) other cannabinoids active at CB_1_, CB_2,_ and/or GPR55 receptors to obtain more intense neuroprotective effects; and (iii) with non-cannabinoid ligands (e.g., adenosine-active ligands) to sum additional therapeutic properties. 

## Data Availability

Data supporting reported results may be supplied upon request to the authors.
